# Association of ultra-processed food consumption with cardiovascular risk factors among patients with type-2 diabetes mellitus

**DOI:** 10.1038/s41387-024-00337-8

**Published:** 2024-10-22

**Authors:** Mohammad Heidari Seyedmahalleh, Ensieh Nasli-Esfahani, Mobina Zeinalabedini, Leila Azadbakht

**Affiliations:** 1 Department of Community Nutrition, School of Nutritional Sciences and Dietetics, Tehran University of Medical Sciences, Tehran, Iran; 2 Students’ Scientific Research Center (SSRC), Tehran University of Medical Sciences (TUMS), Tehran, Iran; 3 Diabetes Research Center, Endocrinology and Metabolism Clinical Sciences Institute, Tehran University of Medical Sciences, Tehran, Iran; 4 Department of Community Nutrition, School of Nutrition and Food Science, Isfahan University of Medical Science, Isfahan, Iran; 5 Department of Clinical Nutrition, School of Nutritional Sciences and Dietetics, Shiraz University of Medical Sciences, Shiraz, Iran

**Keywords:** Diseases, Nutrition

## Abstract

**Background:**

Ultra-processed foods mainly have high energy content and density and low nutrients. Unhealthy lifestyles mainly develop cardiovascular diseases and, as a result, unhealthy food patterns.

**Objective:**

This study aimed to investigate the relationship between the consumption of ultra-processed foods (UPFs) and the risk of novel cardiovascular disease (CVDs) in type-2 diabetes mellitus patients (T2DM).

**Method:**

This is a cross-sectional study that was conducted on 490 type-2 diabetes mellitus patients. A validated 168-item food frequency questionnaire evaluated food intake. Ultra-processed foods were assessed according to NOVA classification. Cardiovascular risk factors such as Castelli risk index 1 and 2 (CRI-I and II), atherogenic index of plasma (AIP), lipid accumulation product (LAP), and cholesterol index (CI) were assessed by traditional CVD risk factors. The anthropometric indices predicting CVD, such as a body shape index (ABSI), body roundness index (BRI), and abdominal volume index (AVI), were assessed.

**Results:**

Each 20-gram increase in UPF consumption was associated with a significant elevation in serum level of TC [B (SE): 1.214 (0.537); 95% CI: 0.159–2.269] and lower HDL serum concentration [B (SE): −0.371 (0.155); 95% CI: −0.675 to −0.067]. The crude model for CRI 1 [B (SE): 0.032 (0.012); 95% CI: 0.009–0.056], CRI 2 [B (SE): 0.022 (0.009); 95% CI: 0.004–0.040], and AIP [B (SE): 0.006 (0.003); 95% CI: 0.000–0.012] showed significant adverse effects.

**Conclusions:**

Our study showed that higher consumption of UPFs is associated with higher chances of developing cardiovascular diseases in T2DM patients.

## Introduction

Type-2 diabetes mellitus (T2DM) is an increasing global health concern [1]. Probable comorbidities are assumed to be correlated with type-2 diabetes, including hypertension, hyperlipidemia, renal dysfunctions, and other related organ failures alongside micro and macrovascular disorders [2, 3]. Individuals with T2DM are highly risky candidates for both micro and macro vascular disorders [4]. Ninety percent of all diabetics are assumed to be T2DM, which accounts for more than 6% of the global population [5]. As the ninth cause of threats against life expectancy, type-2 diabetes mellitus resulted in 1 million deaths in 2017 [6].

Many factors have been suggested as causes of diabetes. Factors such as genetics, abdominal obesity, alcohol consumption, and smoking are among these fields [7]. Meanwhile, unhealthy lifestyles and unhealthy eating habits can be important non-hereditary factors related to the prevalence of diabetes. High energy and low nutrient content, high share of simple carbohydrates, saturated and trans-fat, fewer whole grains, fruits and vegetables, and protein intake are the characteristics of the diet that can be related to the occurrence of diabetes and cardiovascular diseases [8–12].

The Food and Agriculture Organization of the United Nations (FAO) investigated the necessity of research measures in the report “processed foods, quality of Food Intake and health using the NOVA classification system” published in 2019 [13]. Based on this classification, foods under multiple industrial formulations, including sweetened beverages, packaged breads, cakes and cookies, ice creams, breakfast cereals, and frozen meals are in the Ultra-processed food (UPF) classification [14].

Studies declare that most of these products have high energy density and food quality, which means less nutrient density. Consuming more ultra-processed foods is associated with higher chances of diabetes, cardiovascular diseases, hypertension, obesity, and overall diet quality [15–18]. Higher consumption of UPFs was associated with hypertriglyceridemia, lower HDL as well as higher LDL in adults [19]. Similar results were obtained in a study on children, and the consumption of these foods increased total cholesterol and LDL [20]. However, no study has been conducted that has investigated the relationship between the consumption of these foods and the lipid profile among the population of type 2 diabetes patients.

A predictive factor of cardiovascular disease is the blood lipid profile and indicators derived from it [21–23]. Castelli risk index 1 and 2 (CRI 1/2), Atherogenic index of plasma (AIP), and lipid accumulation products (LAP) are novel indicators related to lipid profile and applied to assess the risk of developing CVDs [24–26]. In addition to the previous indices, anthropometric indicators can also help in assessing the risk of cardiovascular diseases [27, 28]. Body shape indices are recent useful parameters accompanying other anthropometric assessing tools to assume the risk of CVDs such as; body shape index (ABSI), abdominal volume index (AVI), and body roundness index (BRI) [29, 30].

Thus, it can be concluded that by correcting eating habits and making healthier food choices, it is possible to help improve weight, and quality of life and reduce the possibility of developing cardiovascular diseases in diabetes. However, no article comprehensively examined the importance of consuming UPFs and their relationship with the occurrence of cardiovascular disease among diabetics. This study uniquely investigates the association between UPF consumption and novel CVD risk factors, specifically in T2DM patients, an understudied high-risk population.

## Method

### Study design and population

This study was conducted according to the guidelines laid down in the Declaration of Helsinki [31]. All procedures involving human patients were performed in accordance with the relevant guidelines and regulations and approved by the Human Ethical Committee of Tehran University of Medical Sciences; [IR.TUMS.MEDICINE.REC.1400.185]. A written informed consent was obtained from all subjects before participating in the project. A total of 490 individuals, aged 35–80, diagnosed with type 2 diabetes, were selected from the Endocrine and Metabolism Research Institute’s diabetes research center between May 2021 and September 2022.

The purpose of this study was to examine the relationship between their consumption of ultra-processed foods (UPFs) and newly identified cardiovascular risk factors using a cross-sectional design.

### Inclusion criteria

Patients who were not on insulin, not pregnant or breastfeeding, not receiving estrogen hormone therapy, not suffering from an autoimmune disease, acute gastrointestinal disorder, acute renal disease, or liver cancer, or whose dietary energy report was either overestimated or underestimated (800 kcal/day to 4200 kcal/day), were not included in the study.

### Data collection

The new cardiovascular risk factors were computed using standard markers such as LDL-C, HDL-C, Chol, and TG. These values were collected from laboratory tests of patients and used in a formula to estimate the new CV risk factors.

Before the initiation of the study, contributors completed written permission forms authorizing that they understood and agreed to participate. Accordingly, the number of participants who did not confirm and complete the ethical consent form was prohibited from participating in the study. Since the patients had to perform biochemical tests for treatment, biochemical indicators such as fasting blood sugar (FBS) and LDL-c were obtained from their medical laboratory test.

$${\rm{CRI}}-{\rm{I}}=\frac{\rm{TC}}{\rm{HDL}-C}$$

$${\rm{CRI}}-{\rm{II}}=\frac{\rm{LDL}-C}{\rm{HDL}-C}$$

$${\rm{LAP}}=\left({\rm{WC}}-65\right)\times \left({\rm{TG}}\right){{\rm{for}}\; {\rm{men}}}$$

$${\rm{LAP}}=\left({\rm{WC}}-58\right)\times \left({\rm{TG}}\right){{\rm{for}}\; {\rm{women}}}$$

$${\rm{CI}}={{\rm{IF}}\; {\rm{TF}}} > 400{\rm{;}}\left({\rm{LDL}}-{\rm{HDL}}+1.5\right)\times {\rm{TG}}$$

$${\rm{CI}}={{\rm{IF}}\; {\rm{TG}}} < 400{\rm{;}}\,({\rm{LDL}}-{\rm{HDL}})\times {\rm{TG}}$$

$${\rm{AIP}}={\rm{Log}}\frac{\rm{TG}}{\rm{HDL}-C}$$

### Demographic and socioeconomic status

A dietician gathered demographic information, such as age, medication history, supplement use, and other data, via patient interviews. We used a socioeconomic status demographic questionnaire to gather information on several aspects such as marital status, education, employment, family size, means of support, method of transportation, and ownership of a private residence. Each item in the questionnaire was assigned codes to determine a socioeconomic status score. This score was obtained by taking the mean of the scores, which ranged from 1 to 10.

### Ultra-processed food intake assessment

An accurate and valid 168-item semi-quantitative food frequency questionnaire (FFQ) was used to estimate typical dietary consumption [32]. A proficient nutritionist gathered nutritional data through a face-to-face interview. Utilizing household measurements, gram consumption was converted to portion sizes. Then, the Nutritionist IV program was used to calculate macro- and micronutrient intakes (First Databank Division, the Hearst Corporation, San Bruno, CA, USA, modified for Iranian foods). To identify highly processed food consumption the NOVA classification [33] was used. Based on this classification, the group “Ultra-processed Foods” is defined as multiple industrial preparations. Out of 168 items considered in our FFQ food list, the following items were assumed ultra-processed foods: biscuits, keraker, cakes (all kinds), canned and conserved foods, burgers, sausages, flavored milk (fruits and chocolate), ice creams, industrial juices, fruit compotes, sauces, jams, soft drinks, industrial and traditional sweets, puffs, cream caramels, industrial and packaged bread, chips, ready-to-eat meals, and pizza. Based on a previous study field [33] as well as a review study [30], we divided all ultra-processed foods into four groups with very few and logical changes: sweetened beverages, sweets, salty snacks, and ultra-processed meats and fast food.

### Anthropometric indices

Patients were weighed while wearing lightweight clothing and without shoes on a digital scale (SECA, Hamburg, Germany) with an accuracy of 0.1 kg. The measurement of standing height was obtained by utilizing a stadiometer that was positioned on the wall. The stadiometer had a high level of precision, accurate to within 0.5 cm. Using a measuring tape, ascertain the waist circumference (WC) of the patient at the place just above the belly button where it is the narrowest. A dietician assessed the hip circumferences of patients by encircling the broadest section of their hips with a tape measure. The body mass index (BMI) was computed using the individual’s height in meters and weight in kilograms. The body mass index (BMI) is computed by dividing an individual’s weight in kilograms by their height in meters squared (kg/m^2^). ABSI, BRI, and abdominal volume index (AVI) were calculated using the procedures outlined earlier, using waist circumference (WC) in meters, body mass index (BMI) in kilograms per square meter, and height in meters, as follows:

$${\rm{ABSI}}=\frac{\rm{WC}}{{\rm{BMI}}^{2/3}\times {\rm{height}}^{1/2}}$$

$${\rm{BRI}}=364.2-365.5\times \sqrt{1-\left(\frac{{\left(\frac{\rm{WC}}{2\pi }\right)}^{2}}{(0.5\,{\rm{height}})^{2}}\right)}$$

$${\rm{AVI}}=\frac{2({\rm{WC}})^{2}+0.7\,\left({\rm{WC}}-{\rm{hip}}\right)^{2}}{1000}$$

### Other variables

The measured level of physical activity was derived from the International Physical Activity Questionnaire (IPAQ) in its brief version. A total of seven questions made up this survey. In all, the questions measured the amount of time spent walking and sitting over the last seven days, as well as the number of days and minutes spent engaging in mild and heavy activities. We thought about using a Five-point MACE in this research. “Major adverse cardiac events” (MACE) include myocardial infarction, stroke, heart failure hospitalization, and revascularization procedures, including angioplasty and bypass surgery. We set it aside since our research sample does not experience CV death. The patients’ medical histories for any chronic conditions were inquired about. The following conditions are part of the patient’s chronic illness history: hypertension, cardiovascular disease, chronic renal disease, chronic obstructive pulmonary disease, and thyroid.

### Statistical analysis

As the first step, we classified subjects based on their daily consumption of UPFs in grams into tertiles. Baseline quantitative and qualitative variables were compared across UPF tertiles by analysis of variance (ANOVA) tests and chi-square, respectively. Continuous variables were reported as mean (SD), and categorical variables were shown as percentages (number). Dietary intakes of overall and four groups of UPF components and some other nutrients were evaluated across UPF tertiles using Analysis of Covariance (ANCOVA). For this purpose, we controlled all food and nutrient intakes for total energy intake (kcal). The associations of UPF tertiles with CVD risk factors and anthropometric indices were explored through linear regression in two different manners. First, we applied a 20-g increase unit in UPF intake. Second, in a further analysis, we used UPF consumption tertile to investigate whether moving towards the highest ranks of UPF consumption has any different associations than the lower ranks. We also implemented binary logistic regression to achieve a more reliable result by comparing them with our main results in linear regression. Based on previous investigations [30], we considered ABSI, BRI, AVI, and AIP cut-off points to be 0.08, 5.20, 17.30, and 0.11, respectively then we transformed these continuous variables into high and low categories to assess their correlation with ultra-processed food consumption tertiles. This categorical representation allowed us to apply binary logistic regression, providing insights into the association between ultra-processed food intake and the categorized outcome variable. This methodological choice aligns with common practices in scientific literature, facilitating a meaningful exploration of the relationship between variables in our analysis of Participants. The same procedure was applied for other CVD risk factors (CRI-1, CRI-2, Cholindex, AIP, and LAP). Odds ratios (ORs) and 95% confidence intervals (95% CIs) were reported for the association of UPF tertiles with CVD risk factors and anthropometric indices in crude and two adjusted models. In the case of anthropometric indices, two multivariable-adjusted models were applied. For the first model, we controlled the confounding effects of energy intake. The second model was adjusted for socioeconomic status, age, sex, and smoking. Table 1 shows which variables significantly differ between tertiles of UPF consumption to choose as confounding variables. The trend of ORs across tertiles of UPF was obtained by considering UPF tertiles as an ordinal variable in the logistic regression models. All statistical analyses were performed by SPSS software (version 26; SPSS et al.). *P* < 0.05 was considered a significant level.

**Table 1:** Descriptive characteristics overall of type-2 diabetes patients across the tertiles of UPF consumption^a^.

Variable	Full sample (*N* = 489)	UPF consumption			*P*-value^b^
		Tertile 1 (lowest) = 163	Tertile 2 = 163	Tertile 3 (highest) = 163	
Age (year)	62.6 (9.8)	65.8 (8.4)	62.6 (9.8)	59.4 (10.4)	<0.000
Sex					
Male (%)	41	25.8	42.3	54.9	<0.000
Female (%)	59	74.2	57.7	45.1	
Welfare	4.7 (1.9)	4.1 (1.8)	4.5 (1.7)	5.5 (1.9)	<0.000
Education (%)					
Below Academic	35.3	48.1	36.2	21.6	<0.000
Academic	64.7	51.9	63.8	78.4	
Marital status (%)					
Married	80.9	80.4	82.2	80.2	0.10
Not Married	6.4	9.8	2.5	6.8	
Divorced	6.1	6.1	6.7	5.6	
Smoking (%)					
Yes	8.8	1.8	5.5	19.1	<0.000
No	91.2	98.2	94.5	80.9	
Physical activity (MET/h)	398.2 (342.1)	392.9 (287.1)	367.9 (282.9)	434.1 (433.8)	0.21
Medication use (%)					
Blood sugar	98.6	98.2	98.8	98.8	0.55
Blood lipid	97.6	97.5	97.5	97.5	0.73
Blood pressure	64.9	63.8	69.3	62	0.46

^a^All values are means (standard deviation (SD)) unless indicated. Ultra-processed foods (UPF) are defined based on NOVA classification for food processing.

^b^Obtained from ANOVA for continuous variables and the chi-square test for categorical variables.

## Results

### Patients’ socio-demographic characteristics

Table 1 shows the sociodemographic of the patient. The participant’s mean age was 62.6 ± 9.8 years, and there were significant differences across the UPF tertiles (*p*-value < 0.001). In the whole population, most of the consumers of UPF were female (59%), but across the tertile, the highest amount was for the first tertile. According to the table, 91.2% of participants were non-smokers, about 98% of them used sugar and lipid-lowering medication, and 65% consumed anti-hypertension drugs. About 80% were married, and most of them were educated.

### Biochemical and anthropometric indices of participants

Table 2 describes patients’ biochemical, novel CVD risk factors, and anthropometric indices. There were significant differences in biochemical and CVD risk factors such as TC (*p*-value = 0.03), CRI-I (*p*-value = 0.03), and CRI-II (*p*-value = 0.05). Also, there was signification in all anthropometric indices except HC (*p*-value = 0.28), and BRI (*p*-value = 0.74).

**Table 2:** Biochemical, cardiometabolic, and anthropometric indices of Type-2 diabetes patients across the tertiles of UPFs^a^.

Variables	UPF consumption				*p*-Value^b^
	Full sample (*N* = 489)	Tertile 1 (lowest) = 163	Tertile 2 = 163	Tertile 3 (highest) = 163	
Biochemical parameters					
FBS (mg/dL)	156.1 (64.4)	156.3 (77.6)	151.4 (55.7)	160.5 (57.6)	0.45
HbA1C (%)	8.0 (1.7)	8.0 (1.9)	7.8 (1.6)	8.2 (1.6)	0.21
HDL (mg/dL)	43.9 (10.6)	44.7 (10.0)	44.3 (11.3)	42.6 (10.3)	0.15
LDL (mg/dL)	77.0 (26.7)	75.4 (25.3)	77.3 (26.8)	78.4 (28.1)	0.58
TC (mg/dL)	143.4 (36.1)	140.1 (31.7)	140.7 (33.7)	149.3 (41.6)	0.03
TG (mg/dL)	141.4 (63.6)	136.3 (58.1)	142.6 (65.1)	145.2 (67.4)	0.43
Anthropometric					
Weight (kg)	73.9 (12.0)	70.3 (11.6)	73.5 (10.4)	77.9 (12.7)	<0.000
BMI (kg/m^2^)	27.5 (4.7)	26.8 (4.2)	27.2 (3.7)	28.5 (5.8)	0.003
WC (cm)	98.2 (9.9)	96.7 (9.8)	98.3 (8.9)	99.5 (10.7)	0.03
HC (cm)	104.1 (9.9)	103.4 (9.9)	103.9 (9.0)	105.1 (10.8)	0.28
ABSI (cm/kg.m)	0.08 (0.006)	0.08 (0.006)	0.08 (0.006)	0.08 (0.006)	0.03
BRI (cm/m)	5.59 (1.21)	5.64 (1.01)	5.54 (1.00)	5.58 (1.54)	0.74
AVI (cm)	19.5 (3.9)	18.9 (3.7)	19.5 (3.4)	20.0 (4.3)	0.03
Cardiometabolic Indices					
AIP	0.12 (0.22)	0.09 (0.21)	0.12 (0.21)	0.14 (0.22)	0.14
LAP	60.16 (33.29)	57.30 (30.86)	61.07 (34.27)	62.14 (34.62)	0.38
CRI 1	3.42 (0.91)	3.30 (0.77)	3.41 (0.95)	3.56 (0.99)	0.03
CRI 2	1.75 (0.70)	1.66 (0.60)	1.74 (0.73)	1.85 (0.74)	0.053
CI	0.86 (0.66)	0.79 (0.60)	0.85 (0.67)	0.93 (0.71)	0.14

^a^All values are means (standard deviation (SD)) unless indicated. Ultra-processed foods (UPF) are defined based on NOVA classification for food processing.

^b^Obtained from ANOVA for continuous variables.

### Nutrients and food groups

Table 3 was designed to show the dietary intake of participants. The number of grams per day and the percentage of energy from the total daily energy intake, respectively, from the first (lowest), second, and third (highest) tertiles are: First: 18.67 g/d, 3.76%; Second: 43.81 g/d, 7.20%; Third: 117.37, 12.94%. These amounts were 59.83 g/d and 7.96% in all cases. the descriptive frequencies manner of main characteristics of the total population in mean (SD) was as follows: Total energy intake: 1643.17 (462.95) kcal/d; values of energy-adjusted dietary that were significantly higher in the third tertile of UPF intake consist of poly-unsaturated fatty acids (P-value = 0.004), Magnesium (*p*-value = 0.012), Calcium (*p*-value = 0.02), sweetened beverages (*p*-value < 0.001), sweets (*p*-value < 0.001), and ultra-processed meats and fast foods (*p*-value < 0.001).

**Table 3:** Energy-adjusted dietary intakes of type-2 diabetes patients across the tertiles of UPFs^a^ consumption^b^.

Dietary intake	UPF consumption			*p*-Value^c^
	Tertile 1 (lowest) (*N* = 163)	Tertile 2 (*N* = 163)	Tertile 3 (highest) (*N* = 161)	
UPF intake (g/day)	18.67 (7.31)	43.81 (9.33)	117.37 (62.81)	<0.000
Total energy intake (kcal/day)	1390.62 (322.83)	1595.00 (310.86)	1945.76 (635.85)	<0.000
UPF energy percent of total energy intake (%)	3.76 (1.68)	7.20 (2.32)	12.94 (5.03)	<0.000
Macronutrients				
UPF (g/d)	35.18 (20.42)	46.65 (21.33)	96.72 (58.14)	<0.000
Protein (g/d)	53.53 (6.31)	52.70 (6.49)	52.05 (7.63)	0.14
Fat (g/d)	61.22 (9.32)	60.42 (9.46)	60.74 (11.15)	0.76
Carbohydrate (g/d)	228.34 (20.41)	231.33 (22.11)	231.40 (24.72)	0.37
Fiber (g/d)	14.62 (2.87)	14.93 (3.05)	14.18 (3.64)	0.10
Main food groups				
Vegetables (g/d)	184.12 (67.49)	197.01 (64.35)	181.39 (76.83)	0.09
Fruits (g/d)	419.5 (119.62)	422.14 (130.59)	411.50 (150.16)	0.76
Red meat (g/d)	18.97 (14.12)	18.40 (10.48)	18.52 (16.41)	0.92
Micronutrients and Trace elements				
SFA (mg/d)	18.16 (3.43)	17.79 (3.97)	18.39 (5.10)	0.43
PUFA (mg/d)	198.9 (104.9)	210.7 (140.04)	247.7 (162.9)	0.004
Iron (mg/d)	12.70 (2.48)	12.86 (2.46)	12.97 (2.78)	0.63
Magnesium (mg/d)	217.55 (29.63)	217.46 (29.61)	208.26 (37.21)	0.01
Zinc (mg/d)	6.53 (1.05)	6.48 (1.03)	6.31 (1.22)	0.19
Chromium (mg/day)	9.1 (6.4)	8.3 (7.2)	9.6 (8.4)	0.23
Calcium (mg/d)	845.98 (212.67)	824.60 (202.35)	781.00 (222.48)	0.02
Vitamin B9 (µ g/d)	250.38 (48.74)	258.75 (54.79)	243.41 (65.79)	0.05
Vitamin B12 (µg/d)	2.84 (0.9)	2.81 (0.8)	2.68 (0.9)	0.25
Vitamin D (µ g/d)	4.34 (1.10)	4.34 (1.21)	4.20 (0.91)	0.42
Vitamin K (µ g/d)	127.50 (44.76)	132.97 (45.70)	126.30 (55.93)	0.42
Vitamin C (mg/d)	132.65 (34.09)	140.74 (38.05)	133.51 (42.55)	0.11
Main UPF groups				
Sweetened beverages (g/day)	4.89 (5.17)	6.51 (8.91)	31.92 (41.72)	<0.000
Sweets (g/day)	21.85 (12.34)	28.72 (15.14)	50.64 (31.33)	<0.000
Salty snacks (g/day)	2.07 (2.59)	1.60 (2.70)	7.03 (47.81)	0.14
Ultra-processed meats and fast food (g/day)	4.81 (4.14)	7.16 (7.47)	12.67 (0.99)	<0.000

^a^Ultra-processed foods (UPF) defined based on NOVA classification for food processing.

^b^All values are means ± standard error (SE); total energy and macronutrient intake are adjusted for age and gender; all other values are adjusted for age, gender, and energy intake.

^c^Obtained from ANCOVA.

### Biochemical and anthropometric indices

The linear association of each 20-g increase in UPF consumption with Biochemical and anthropometric is represented in Table 4. In the fully adjusted model, each 20-g increase in UPF consumption was associated with a significant elevation in serum level of TC [B (SE): 1.214 (0.537); 95% CI: 0.159–2.269]. Also, lower HDL serum concentration was related to an increase in UPF intake [B (SE): −0.371 (0.155); 95% CI: −0.675 to −0.067], although this relationship was not significant in the fully adjusted model [B (SE): −0.237 (0.154); 95% CI: −0.540 to 0.066]. Other indices, including BMI, WC, HC, FBS, TG, and LDL, did not show any significant association with each 20 g increase in UPF consumption.

**Table 4:** Linear regression association between each 20 g increase in UPF consumption and anthropometric and biochemical level.

Outcome	UPF		95% CI^a^
	B (SE)^a^	*p*-Value^a^	
BMI			
Crude^b^	0.046 (0.062)	0.457	−0.076 to 0.168
Model 1^c^	0.008 (0.069)	0.910	−0.129 to 0.144
Model 2^d^	0.001 (0.070)	0.989	−0.136 to 0.138
WC			
Crude^b^	0.079 (0.130)	0.542	−0.175 to 0.334
Model 1^c^	0.093 (0.146)	0.523	−0.193 to 0.379
Model 2^d^	0.104 (0.146)	0.477	−0.183 to 0.390
HC			
Crude^b^	−0.042 (0.130)	0.747	−0.298 to 0.214
Model 1^c^	0.003 (0.146)	0.986	−0.285 to 0.290
Model 2^d^	0.043 (0.142)	0.762	−0.235 to 0.321
FBS			
Crude^b^	1.051 (0.841)	0.212	−0.601 to 2.703
Model 1^c^	1.025 (0.944)	0.278	−0.831 to 2.880
Model 2^d^	0.515 (0.983)	0.601	−1.417 to 2.446
TC			
Crude^b^	1.333 (0.469)	0.005	0.412−2.255
Model 1^c^	1.237 (0.527)	0.019	0.202−2.272
Model 2^d^	1.214 (0.537)	0.024	0.159−2.269
TG			
Crude^b^	0.708 (0.832)	0.395	−0.926 to 2.343
Model 1^c^	0.399 (0.934)	0.669	−1.436 to 2.234
Model 2^d^	−0.019 (0.955)	0.984	−1.895 to 1.857
HDL			
Crude^b^	−0.394 (0.138)	0.004	−0.665 to −0.123
Model 1^c^	−0.371 (0.155)	0.017	−0.675 to −0.067
Model 2^d^	−0.237 (0.154)	0.125	−0.540 to 0.066
LDL			
Crude^b^	0.076 (0.350)	0.828	−0.612 to 0.764
Model 1^c^	−0.369 (0.391)	0.346	−1.136 to 0.399
Model 2^d^	−0.424 (0.403)	0.293	−1.216 to 0.368

^a^Calculated using linear regression presented as beta (SE), *p*-value, and 95% CI for the significance of findings.

^b^Crude: Not adjusted for any variables.

^c^Model adjusted for energy intake.

^d^Model adjusted for energy intake plus socioeconomic status, age, sex, and smoking.

### Novel anthropometric and lipid indices

According to Table 5, which declares the linear regression relationship between each 20-g increase in UPF consumption and novel cardiovascular indices, as the intake of UPF elevates, almost none of the indices significantly change. However, only in the crude model for three indices slight change though statistically significant was observed. CRI 1 [B (SE): 0.032 (0.012); 95% CI: 0.009 – 0.056], CRI 2 [B (SE): 0.022 (0.009); 95% CI: 0.004–0.040], and AIP [B (SE): 0.006 (0.003); 95% CI: 0.000–0.012]. These findings show that the increase in UPF intake is mostly correlated with blood cholesterol rather than other cardiovascular-related indices.

**Table 5:** Linear regression association between each 20 g increase in UPF consumption and novel CVD risk factors and anthropometrics reporting in beta, *p*-value, and 95% confidence interval.

Outcome	UPF		95% CI^a^
	B (SE)^a^	*p*-Value^a^	
CRI 1			
Crude^b^	0.032 (0.012)	0.007	0.009 to 0.056
Model 1^c^	0.021 (0.013)	0.107	−0.005 to 0.048
Model 2^d^	0.009 (0.014)	0.526	−0.018 to 0.035
CRI 2			
Crude^b^	0.022 (0.009)	0.014	0.004 to 0.040
Model 1^c^	0.013 (0.010)	0.207	−0.007 to 0.033
Model 2^d^	0.005 (0.010)	0.637	−0.016 to 0.026
Cholesterol index			
Crude^b^	0.013 (0.009)	0.133	−0.004 to 0.030
Model 1^c^	0.001 (0.010)	0.922	−0.018 to 0.020
Model 2^d^	−0.004 (0.010)	0.703	−0.024 to 0.016
AIP			
Crude^b^	0.006 (0.003)	0.034	0.000 to 0.012
Model 1^c^	0.006(0.003)	0.077	−0.001 to 0.012
Model 2^d^	0.003 (0.003)	0.379	−0.004 to 0.009
LAP			
Crude^b^	0.115 (0.435)	0.792	−0.740 to 0.970
Model 1^c^	0.070 (0.489)	0.886	−0.890 to 1.030
Model 2^d^	0.113 (0.493)	0.819	−0.855 to 1.081
AVI			
Crude^b^	0.032 (0.051)	0.538	−0.069 to 0.132
Model 1^c^	0.038 (0.058)	0.505	−0.075 to 0.152
Model 2^d^	0.046 (0.058)	0.429	−0.068 to 0.160
BRI			
Crude^b^	−0.026 (0.016)	0.099	−0.057 to 0.005
Model 1^c^	−0.007 (0.018)	0.693	−0.042 to 0.028
Model 2^d^	0.015 (0.016)	0.356	−0.017 to 0.047
ABSI			
Crude^b^	0.000 (0.000)	0.258	0.000 to 0.000
Model 1^c^	0.000 (0.000)	0.598	0.000 to 0.000
Model 2^d^	0.000 (0.000)	0.148	0.000 to 0.000

^a^Calculated using linear regression presented as Beta (SE), *p*-value, and 95% CI for the significance of findings.

^b^Crude: Not adjusted for any variables.

^c^Model adjusted for energy intake.

^d^Model adjusted for energy intake plus socioeconomic status, age, sex, and smoking.

We implemented further analysis and applied UPF consumption in tertiles of daily intake instead of each 20-g unit increase. As is presented in Table 6, comparing the third tertile (highest intake) to the second and first tertile (lowest intake) it is noticeable that in most of the indices, the linear regression relationship is significant even the ones with non-significant relation in the previous analysis. As an example, anthropometric indices like AVI, which showed no obvious and significant effect from each 20-g increase in UPF intake, here have significantly increased by moving from the first tertile towards the third tertile of UPF consumption [B (SE): 0.736 (0.247); *p*-value = 0.003]. On the other hand, despite CI not demonstrating a significant relationship with UPF consumption in Table 5., in this analysis it is declared that by reaching a higher tertile of UPF grams daily intake a significant increase in CI is investigated B (SE): 0.070 (0.037); *p*-value = 0.056].

**Table 6:** Linear regression association between UPF consumption tertiles and novel CVD risk factors and anthropometrics reporting in Beta (standard error) and *p*-value.

Outcome	UPF tertiles		
	B^a^	SE^a^	*p*-Value^a^
CRI 1	0.126	0.050	0.012
Energy adjusted model	0.074	0.058	0.201
CRI 2	0.091	0.039	0.019
Energy adjusted model	0.047	0.044	0.289
Cholesterol index	0.070	0.037	0.056
Energy adjusted model	0.018	0.042	0.662
Atherogenic index of plasma (AIP)	0.024	0.012	0.053
Energy adjusted model	0.022	0.014	0.121
Lipid accumulation products (LAP)	2.336	1.842	0.205
Energy adjusted model	2.714	2.113	0.200
AVI	0.565	0.216	0.009
Energy adjusted model	0.736	0.247	0.003
BRI	-0.032	0.067	0.630
Energy adjusted model	0.081	0.076	0.287
ABSI	-0.001	0.000	0.021
Energy adjusted model	0.000	0.000	0.492

^a^Calculated using linear regression presented as beta (SE) and *p*-value for significance of findings.

As shown in Supplementary Table 1, binary logistic regression was performed, too, and as a result, some of the outcome findings were different from the linear regression analyses. For instance, LAP which did not show significant association in any of the analyses, through logistic regression in all models, a considerable and significant relation was found [OR (95% CI): 2.20 (1.23–3.93); P-trend = 0.008]

## Discussion

This study was designed and implemented to evaluate the relationship between consuming ultra-processed foods and the risk of cardiovascular diseases in type-2 diabetic patients. This study shows a direct association between high consumption of UPFs and CVD risk factors and anthropometrics-related CVD risk factors. High consumers of UPFs had about two times more odds of high LAP, ABSI, BRI, and AVI [13, 15, 18, 34].

This study found significant associations between the consumption of UPFs and most of the CVD indicators. These indicators are based on blood cholesterol levels and vascular blocking factors. In similar studies, the results agree with our findings. For instance, in a longitudinal study in Brazil that was conducted on children, higher consumption of UPFs was associated with increased total and LDL cholesterol compared to the beginning of the study [20]. Published data from prospective cohort studies support our findings too. Nutri Net-Santé cohort on adults in France and UK Biobank Cohort on above 40-year-old participants showed that for each unit increase in UPF share of daily energy, the risk of overall CVD increased by 12% and 17%, respectively [35, 36].

The findings of our study show that the average daily intake of energy from UPF is about 8% of the total energy in the overall population and 13% in the highest consumer tertile. These numbers are much lower than the average consumption in most developed European and American countries. They are almost equal to the amount of consumption in countries with minimal consumption, such as the Mediterranean and Asian countries. However, the statistical correlation with CVD is more similar to countries with high consumption, such as France, England, and America [37, 38]. In a study conducted on Iranian children, the amount of UPF consumption was about 60%, which had no significant association with obesity and overweight [39]. Together, these show that UPF consumption is more common at younger ages, as there was a significant age difference between tertiles of consumption in our study (*p*-value < 0.001). Also, this fact can be related to Iranians’ less adherence to a healthy diet such as the Mediterranean diet, which is more effective in older ages. As shown in Table 2, the consumption of fruits, vegetables, and fiber, controlled for energy, did not differ significantly among the UPF consumption groups and was less than the recommended amount, the same as most other vital minerals and vitamins.

The daily intake of saturated fatty acids is highly related to the incidence of CVD. According to the latest guidelines published by the American Heart Association, the permissible daily amount of SFA is 5–6% of daily calories [33, 40], while this amount is about 18% among the participants of our study, and there is no significant difference between the groups (*p*-value = 0.43). In general, in addition to being associated with a higher risk of CVD, the consumption of more UPFs, along with other components of an unhealthy diet, leads to a higher risk.

Blood lipid profile, except for TC and HDL was mainly uninfluenced by an increase in UPF consumption in linear analysis. This can be mostly since, in general, the lipid profile of patients with diabetes due to drug treatments and the history of accompanying chronic diseases are impaired [41], thus the differences and statistical significance were not found and this is more justified when according to Table 1, almost all of the participants were on blood lipid and sugar controlling medication and there were no significant between-group differences (*p*-value = 0.55)

Our findings underscore the significant impact of UPF consumption on key biochemical and anthropometric indices associated with cardiovascular disease risk. Specifically, each 20-g increase in UPF intake was positively correlated with elevated serum total cholesterol levels (TC) and decreased high-density lipoprotein (HDL) concentration, indicative of adverse lipid profiles commonly associated with cardiovascular disease risk. These associations remained robust even after adjustments for potential confounders, highlighting the independent influence of UPF consumption on lipid metabolism.

Interestingly, while UPF consumption did not exhibit significant associations with conventional anthropometric indices such as body mass index (BMI), waist circumference (WC), hip circumference (HC), fasting blood sugar (FBS), triglycerides (TG), and low-density lipoprotein (LDL), our analysis revealed notable shifts in novel anthropometric and lipid indices. Although the overall changes were subtle, certain indices, such as cardiovascular risk index 1 (CRI 1), cardiovascular risk index 2 (CRI 2), and atherogenic index of plasma (AIP), demonstrated statistically significant alterations in response to UPF intake, particularly in the crude model.

**Fig. 1:**
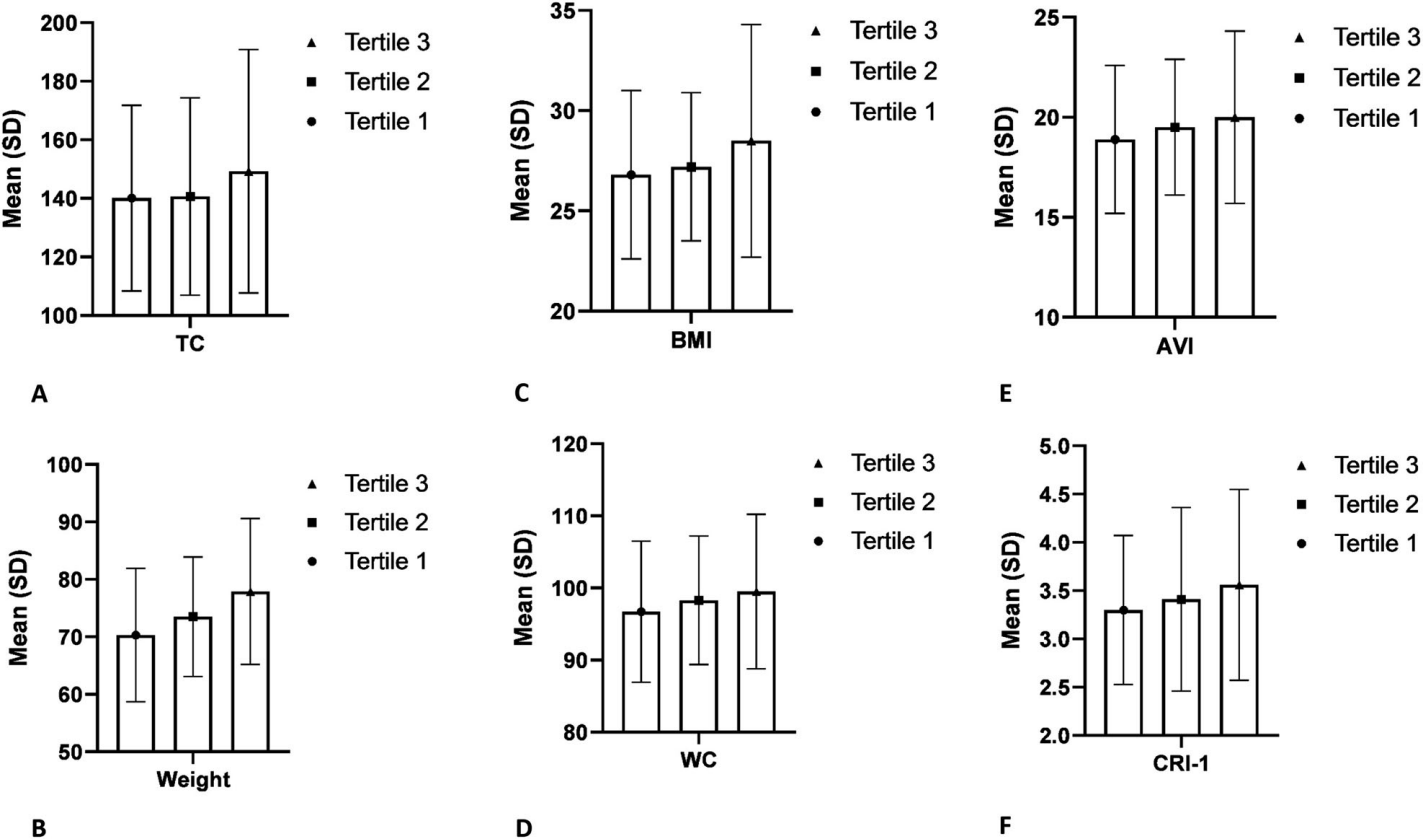
Mean values of selected variables among tertiles of UPF consumption. Data obtained from ANOVA analyses and presented as mean and standard deviation. All findings represent statistically significant differences between UPF tertiles (*P* value < 0.05). **A** TC; **B** weight; **C** BMI; **D** WC; **E** AVI; **F** CRI-1.

**Fig. 2:**
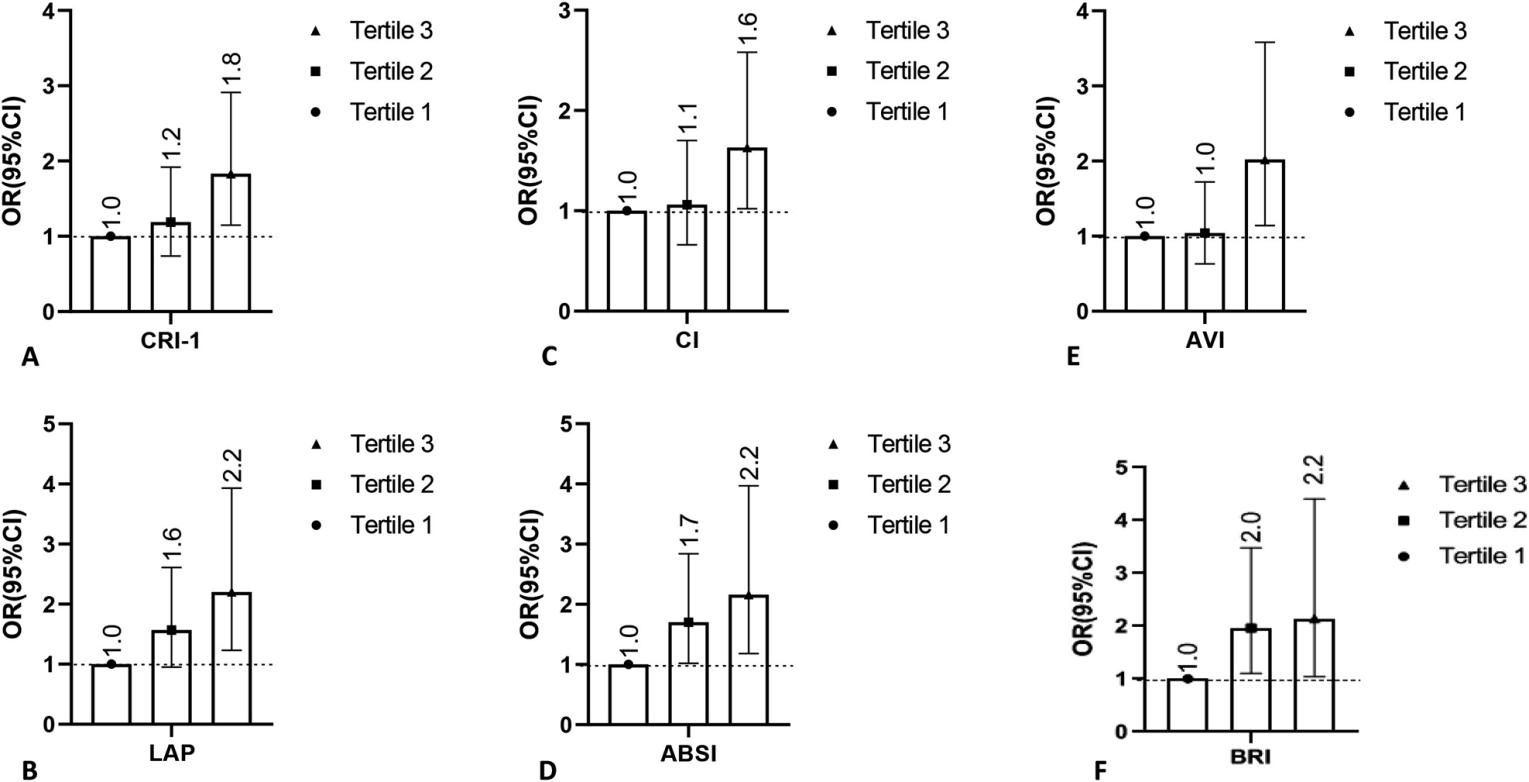
Odds of developing abnormalities for selected CVD risk factor indices across the tertiles of UPF AQ6 consumption. Data obtained from logistic regression analyses and represented as odds ratio with 95% confidence interval. Interpreting that all findings presented here are statistically significant since the number 1 was not included in the confidence interval. **A** CRI-1; **B** LAP, **C** CI; **D** ABSI; **E** AVI; **F** BRI.

Further exploration through tertile analysis revealed a dose–response association between UPF consumption and cardiovascular risk indices, with individuals in the highest tertile exhibiting more pronounced changes compared to those in the lowest tertile. Notably, indices previously unaffected by incremental UPF intake, such as abdominal volume index (AVI) and cardiac index (CI), showed significant associations in this analysis, underscoring the importance of considering cumulative intake levels when evaluating cardiovascular risk. (Figs. 1 and 2). This finding opposes the results of other studies, which have observed that more consumption of UPFs is significantly associated with higher BMI and waist circumference (WC) as part of the indicators that determine body shape [18, 42].

Moreover, logistic regression analysis provided additional insights, revealing significant associations between UPF consumption and certain indices that were not evident in linear regression models. The observed discrepancy suggests that the impact of UPF consumption on cardiovascular risk may be influenced by the sensitivity of the analytical approach to differences in participant characteristics. Linear regression assumes that the outcome variable is normally distributed and that the relationship between the predictor and outcome is linear [43]. In the case of this study, linear regression may have failed to capture certain associations between UPF consumption and cardiovascular risk indices, particularly if the relationships were non-linear or if there were influential outliers in the data.

Most of these foods have high energy content and density along with low nutritional content. For example, refined carbohydrates and saturated and trans fats in these foods can explain the association between consuming more UPFs and abdominal obesity and the accumulation of visceral fat by increasing the glycemic response and insulin hormone secretion and reducing the body’s sensitivity to it [44–47]. Also, the content of fiber and minerals are greatly reduced due to processing and also the difficulty of packaging fresh products like fruits and vegetables [48, 49].

**Fig. 3:**
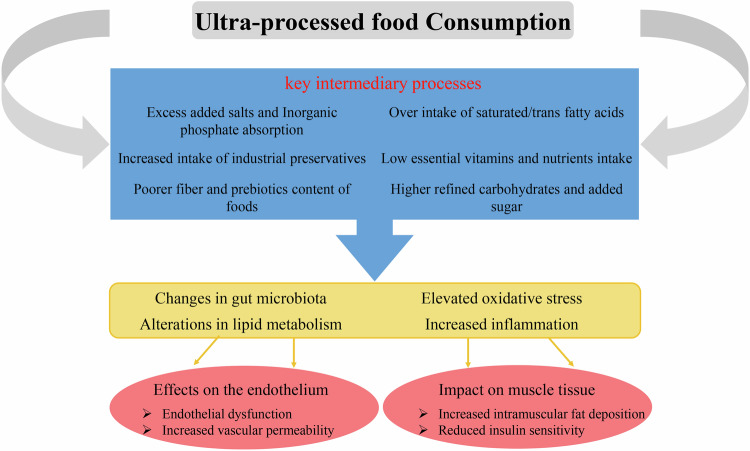
Mechanism and biological pathways through which consuming more UPFs leads to adverse health outcomes. Higher consumption of UPFs leads to changes in immune system, microbiota, and metabolism through various intermediary processes and causes further health damages to human health.

The relationship between the consumption of ultra-processed foods and high-risk cardiovascular outcomes has been confirmed by cellular and molecular studies. These effects have been modeled in cases such as increasing the GI/GL of food, increasing the concentration of inflammatory and oxidative compounds during the processing process, disturbing the intestinal microbial flora and energy-mediated pathways such as action failure of neural and chemical messengers of digestion and absorption like; ghrelin and PYY [48]. As has been emphasized so far, the relationship between the consumption of UPFs is not unique to the type of these foods and their contents, but their relationship with receiving more energy and their unhealthy lifestyle increases the risk of disease in such a way that controlling its indirect effects in Statistical analysis caused many changes in our findings. Another unhealthy consequence of consuming higher UPFs is attributed to their destructive effects on endothelium structure and function [48]. Studies have shown that diets containing more industrially processed foods with unhealthy amounts of added slats lead to a greater amount of circulating phosphorus in the body and, accordingly, may result in an increase in tissue and serum phosphate, which is associated with elevated oxidative stress of the endothelial cells and abruption in function [50, 51]. Moreover, UPFs, as foods rich in trans fatty acids (TFAs), can impair the normal function of endothelial cells by increasing LDL and decreasing HDL [52]. The disadvantages of dedicating more of daily calorie intake to UPFs are expanded to more dysfunctions like accumulation of advanced glycation end-products (AGEs) and impaired mitochondrial function, alteration in proliferation, apoptosis, and signaling pathways of immune cells, escalated reactive oxygen species production, and malfunction in the immune system and response to infections [53, 54] (Fig. 3).

To the best of our knowledge, this was the first study to explore the link between UPF consumption and CVD risk factors in T2DM patients. Our findings align with evidence in this area. A preliminary study by Zeinalabedini et al. found that higher scores of HEI-2015 were associated with greater odds of developing unhealthy AIP and BRI in the current population [55]. However, their results failed to observe any associations with ABSI, AVI and TC. On the other hand, we investigated the linear associations helping to find dose-dependent links and interpreting novel findings in this area. Moreover, HEI is mostly dependent on nine beneficial and four food groups in moderation [56, 57], while UPFs, according to NOVA classification, originate from a more detailed classification focusing on nutrient quality along with industrial processing on food causing chemical and textural alterations on products [13]. Another study investigated the links between UPF and CVD risk factors in obese and overweight women and, in contrast with our findings, found no significant association with anthropometric nor with blood lipid risk factors [58]. Another cross-sectional study performed on Iranian adults revealed a significant inverse association between UPF consumption and TG and HDL but not with TC or anthropometric risk factors [59]. Moreover, a review study conducted in 2020 observed that higher UPF intake is associated with adverse anthropometric outcomes like weight, WC, and BMI, along with biochemical indices like HDL [18]. Using new tools to predict cardiovascular diseases is one of the innovations and strengths of our study. In addition, before this, the relationship between the consumption of ultra-processed foods and the risk of CVDs in type 2 diabetes patients had not been measured, and few studies had investigated the relationship between the consumption of these foods and lipid profile indicators. Moreover, we applied both logistic and linear regression analyses for more valid and more reliable findings. However, there were some limitations in our work, such as the fact that most of the patients, due to eating habits caused by disease and age, did not consume much UPFs, although there was a significant difference in statistical analysis among the groups. Also, the lipid profile of the patients was affected by drug therapy, so no significant results were observed.

## Conclusion

Our study showed that the consumption of UPFs, which are mainly part of unhealthy food groups, is related to abdominal obesity and more unfavorable anthropometric results among patients with type 2 diabetes, the same as some of the indicators related to blood lipids like LAP. In general, reducing the consumption of UPFs can be a good choice to reduce the risk of developing cardiovascular diseases. Further studies may be required to assess the longitudinal associations in addition to the direction of association and casualty through prospective study designs to better determine these associations and confirm the findings in the present study.

## Supplementary information

Multivariable-adjusted odds ratio novel CVD risk factors and anthropometrics across tertiles of UPFs consumption

## Data Availability

The data sets used and/or analyzed during the current study are available from the corresponding author upon reasonable request.

## References

[1] Olokoba AB, Obateru OA, Olokoba LB. Type 2 diabetes mellitus: a review of current trends. Oman Med J. 2012;27:269. 23071876 10.5001/omj.2012.68 PMC3464757

[2] DeFronzo RA, Ferrannini E, Groop L, Henry RR, Herman WH, Holst JJ, et al. Type 2 diabetes mellitus. Nat Rev Dis Prim. 2015;1:15019. 27189025 10.1038/nrdp.2015.19

[3] Pinhas-Hamiel O, Zeitler P. Acute and chronic complications of type 2 diabetes mellitus in children and adolescents. Lancet. 2007;369:1823–31. 17531891 10.1016/S0140-6736(07)60821-6

[4] DeFronzo RA, Ferrannini E, Groop L, Henry RR, Herman WH, Holst JJ, et al. Type 2 diabetes mellitus. Nat Rev Dis Prim. 2015;1:1–22. 10.1038/nrdp.2015.19 27189025

[5] Janka H-U, Michaelis D. Epidemiology of diabetes mellitus: prevalence, incidence, pathogenesis, and prognosis. Z Arztl Fortbild Qualitatssich. 2002;96:159–65. 12017759

[6] Khan MAB, Hashim MJ, King JK, Govender RD, Mustafa H, Al Kaabi J. Epidemiology of type 2 diabetes–global burden of disease and forecasted trends. J Epidemiol Glob Health. 2020;10:107. 32175717 10.2991/jegh.k.191028.001 PMC7310804

[7] Kohei K. Pathophysiology of type 2 diabetes and its treatment policy. JMAJ. 2010;53:41–6.

[8] De Koning L, Chiuve SE, Fung TT, Willett WC, Rimm EB, Hu FB. Diet-quality scores and the risk of type 2 diabetes in men. Diabetes Care. 2011;34:1150–6. 21464460 10.2337/dc10-2352 PMC3114491

[9] Fung TT, McCullough M, Van Dam RM, Hu FB. A prospective study of overall diet quality and risk of type 2 diabetes in women. Diabetes Care. 2007;30:1753–7. 17429059 10.2337/dc06-2581

[10] Ley SH, Pan A, Li Y, Manson JE, Willett WC, Sun Q, et al. Changes in overall diet quality and subsequent type 2 diabetes risk: three US prospective cohorts. Diabetes Care. 2016;39:2011–8. 27634391 10.2337/dc16-0574 PMC5079614

[11] McNaughton SA, Dunstan DW, Ball K, Shaw J, Crawford D. Dietary quality is associated with diabetes and cardio-metabolic risk factors. J Nutr. 2009;139:734–42. 19211825 10.3945/jn.108.096784

[12] Xu Z, Steffen LM, Selvin E, Rebholz CM. Diet quality, change in diet quality and risk of incident CVD and diabetes. Public Health Nutr. 2020;23:329–38. 31511110 10.1017/S136898001900212X PMC6992481

[13] Monteiro CA. Nutrition and health. The issue is not food, nor nutrients, so much as processing. Public Health Nutr. 2009;12:729–31. 19366466 10.1017/S1368980009005291

[14] Monteiro CA, Cannon G, Moubarac J-C, Levy RB, Louzada MLC, Jaime PC. The UN decade of nutrition, the NOVA food classification and the trouble with ultra-processing. Public Health Nutr. 2018;21:5–17. 28322183 10.1017/S1368980017000234 PMC10261019

[15] Lane MM, Davis JA, Beattie S, Gómez‐Donoso C, Loughman A, O’Neil A, et al. Ultraprocessed food and chronic noncommunicable diseases: a systematic review and meta‐analysis of 43 observational studies. Obes Rev. 2021;22:e13146. 33167080 10.1111/obr.13146

[16] Martini D, Godos J, Bonaccio M, Vitaglione P, Grosso G. Ultra-processed foods and nutritional dietary profile: a meta-analysis of nationally representative samples. Nutrients. 2021;13:3390. 34684391 10.3390/nu13103390 PMC8538030

[17] Moradi S, Bagheri R, Mohammadi H, Jayedi A, Lane MM, Asbaghi O, et al. Ultra-processed food consumption and adult diabetes risk: a systematic review and dose-response meta-analysis. Nutrients. 2021;13:4410. 34959961 10.3390/nu13124410 PMC8705763

[18] Pagliai G, Dinu M, Madarena M, Bonaccio M, Iacoviello L, Sofi F. Consumption of ultra-processed foods and health status: a systematic review and meta-analysis. Br J Nutr. 2021;125:308–18. 32792031 10.1017/S0007114520002688 PMC7844609

[19] Donat-Vargas C, Sandoval-Insausti H, Rey-García J, Moreno-Franco B, Åkesson A, Banegas JR, et al. High consumption of ultra-processed food is associated with incident dyslipidemia: a prospective study of older adults. J Nutr. 2021;151:2390–8. 34038538 10.1093/jn/nxab118

[20] Rauber F, Campagnolo PD, Hoffman DJ, Vitolo MR. Consumption of ultra-processed food products and its effects on children’s lipid profiles: a longitudinal study. Nutr Metab Cardiovasc Dis. 2015;25:116–22. 25240690 10.1016/j.numecd.2014.08.001

[21] Castelli WP, Wilson PWF, Levy D, Anderson K. Cardiovascular risk factors in the elderly. Am J Cardiol. 1989;63:12–9. 10.1016/0002-9149(89)90110-0 2523187

[22] Wood D. Established and emerging cardiovascular risk factors. Am Heart J. 2001;141:S49–57. 11174359 10.1067/mhj.2001.109951

[23] Abourbih S, Filion KB, Joseph L, Schiffrin EL, Rinfret S, Poirier P, et al. Effect of fibrates on lipid profiles and cardiovascular outcomes: a systematic review. Am J Med. 2009;122:962.e1–8. 19698935 10.1016/j.amjmed.2009.03.030

[24] Bhardwaj S, Bhattacharjee J, Bhatnagar M, Tyagi S, Delhi N. Atherogenic index of plasma, castelli risk index and atherogenic coefficient-new parameters in assessing cardiovascular risk. Int J Pharm Biol Sci. 2013;3:359–64.

[25] Koleva DI, Andreeva-Gateva PA, Orbetzova M, Atanassova I, Nikolova J. Atherogenic index of plasma, castelli risk indexes and leptin/adiponectin ratio in women with metabolic syndrome. Int J Pharm Med Res. 2015;3:12–6.

[26] Salcedo-Cifuentes M, Belalcazar S, Acosta EY, Medina-Murillo JJ. Conventional biomarkers for cardiovascular risks and their correlation with the castelli risk index-indices and TG/HDL-c. Arch Med. 2020;20:11–22.

[27] Ho SC, Chen YM, Woo JLF, Leung SSF, Lam TH, Janus ED. Association between simple anthropometric indices and cardiovascular risk factors. Int J Obes. 2001;25:1689–97. 10.1038/sj.ijo.0801784 11753592

[28] Huang KC, Lin WY, Lee LT, Chen CY, Lo H, Hsia HH, et al. Four anthropometric indices and cardiovascular risk factors in Taiwan. Int J Obes. 2002;26:1060–8. 10.1038/sj.ijo.0802047 12119571

[29] Maessen MFH, Eijsvogels TMH, Verheggen RJHM, Hopman MTE, Verbeek ALM, de Vegt F. Entering a new era of body indices: the feasibility of a body shape index and body roundness index to identify cardiovascular health status. PLoS ONE. 2014;9:e107212. 25229394 10.1371/journal.pone.0107212 PMC4167703

[30] Mameli C, Krakauer NY, Krakauer JC, Bosetti A, Ferrari CM, Moiana N, et al. The association between a body shape index and cardiovascular risk in overweight and obese children and adolescents. PLoS ONE. 2018;13:e0190426. 29298340 10.1371/journal.pone.0190426 PMC5752028

[31] Association WM. World Medical Association Declaration of Helsinki: ethical principles for medical research involving human subjects. J Am Med Assoc. 2013;310:2191–4. 10.1001/jama.2013.281053 24141714

[32] Mirmiran P, Hosseini Esfahani F, Mehrabi Y, Hedayati M, Azizi F. Reliability and relative validity of an FFQ for nutrients in the Tehran Lipid and Glucose Study. Public Health Nutr. 2010;13:654–62. 19807937 10.1017/S1368980009991698

[33] Eckel RH, Jakicic JM, Ard JD, de Jesus JM, Miller NH, Hubbard VS, et al. 2013 AHA/ACC guideline on lifestyle management to reduce cardiovascular risk: a report of the American College of Cardiology/American Heart Association Task Force on Practice Guidelines. Circulation. 2014;129:S76–99. 24222015 10.1161/01.cir.0000437740.48606.d1

[34] Haddad L, Hawkes C, Waage J, Webb P, Godfray C, Toulmin C, et al. Food systems and diets: facing the challenges of the 21st century. Global Panel on Agriculture and Food Systems for Nutrition. 2016.

[35] Srour B, Fezeu LK, Kesse-Guyot E, Allès B, Méjean C, Andrianasolo RM, et al. Ultra-processed food intake and risk of cardiovascular disease: prospective cohort study (NutriNet-Santé). BMJ (Clin Res Ed). 2019;365:l1451. 10.1136/bmj.l1451 PMC6538975 31142457

[36] Chen X, Chu J, Hu W, Sun N, He Q, Liu S, et al. Associations of ultra-processed food consumption with cardiovascular disease and all-cause mortality: UK Biobank. Eur J public health. 2022;32:779–85. 36006020 10.1093/eurpub/ckac104 PMC9527958

[37] Marino M, Puppo F, Del Bo’ C, Vinelli V, Riso P, Porrini M, et al. A systematic review of worldwide consumption of ultra-processed foods: findings and criticisms. Nutrients 2021;13:2778. 34444936 10.3390/nu13082778 PMC8398521

[38] Baker P, Machado P, Santos T, Sievert K, Backholer K, Hadjikakou M, et al. Ultra‐processed foods and the nutrition transition: global, regional and national trends, food systems transformations and political economy drivers. Obes Rev. 2020;21:e13126. 32761763 10.1111/obr.13126

[39] Asgari E, Askari M, Bellissimo N, Azadbakht L. Association between ultraprocessed food intake and overweight, obesity, and malnutrition among children in Tehran, Iran. Int J Clin Pract. 2022;2022:8310260. 10.1155/2022/8310260 PMC9433238 36081808

[40] Briggs MA, Petersen KS, Kris-Etherton PM, editors. Saturated fatty acids and cardiovascular disease: replacements for saturated fat to reduce cardiovascular risk. Healthcare; 2017: MDPI. 10.3390/healthcare5020029 PMC5492032 28635680

[41] Khan H, Sobki S, Khan S. Association between glycaemic control and serum lipids profile in type 2 diabetic patients: HbA 1c predicts dyslipidaemia. Clin Exp Med. 2007;7:24–9. 17380302 10.1007/s10238-007-0121-3

[42] Askari M, Heshmati J, Shahinfar H, Tripathi N, Daneshzad E. Ultra-processed food and the risk of overweight and obesity: a systematic review and meta-analysis of observational studies. Int J Obes. 2020;44:2080–91. 10.1038/s41366-020-00650-z 32796919

[43] Austin PC, Steyerberg EW. The number of subjects per variable required in linear regression analyses. J Clin Epidemiol. 2015;68:627–36. 25704724 10.1016/j.jclinepi.2014.12.014

[44] Dicken SJ, Batterham RL. The role of diet quality in mediating the association between ultra-processed food intake, obesity and health-related outcomes: a review of prospective cohort studies. Nutrients. 2021;14:23. 35010898 10.3390/nu14010023 PMC8747015

[45] Konieczna J, Morey M, Abete I, Bes-Rastrollo M, Ruiz-Canela M, Vioque J, et al. Contribution of ultra-processed foods in visceral fat deposition and other adiposity indicators: prospective analysis nested in the PREDIMED-Plus trial. Clin Nutr. 2021;40:4290–300. 33610419 10.1016/j.clnu.2021.01.019

[46] Siri-Tarino PW, Chiu S, Bergeron N, Krauss RM. Saturated fats versus polyunsaturated fats versus carbohydrates for cardiovascular disease prevention and treatment. Annu Rev Nutr. 2015;35:517–43. 26185980 10.1146/annurev-nutr-071714-034449 PMC4744652

[47] Zamanillo-Campos R, Chaplin A, Romaguera D, Abete I, Salas-Salvadó J, Martín V, et al. Longitudinal association of dietary carbohydrate quality with visceral fat deposition and other adiposity indicators. Clin Nutr. 2022;41:2264–74. 36084360 10.1016/j.clnu.2022.08.008 PMC9529821

[48] Juul F, Vaidean G, Parekh N. Ultra-processed foods and cardiovascular diseases: potential mechanisms of action. Adv Nutr. 2021;12:1673–80. 33942057 10.1093/advances/nmab049 PMC8483964

[49] Valicente VM, Peng C-H, Pacheco KN, Lin L, Kielb EI, Dawoodani E, et al. Ultra-processed foods and obesity risk: a critical review of reported mechanisms. Adv Nutr. 2023;14:718–38. 10.1016/j.advnut.2023.04.006 PMC10334162 37080461

[50] Calvo MS, Uribarri J. Public health impact of dietary phosphorus excess on bone and cardiovascular health in the general population. Am J Clin Nutr. 2013;98:6–15. 23719553 10.3945/ajcn.112.053934

[51] Tzemos N, Lim PO, Wong S, Struthers AD, MacDonald TM. Adverse cardiovascular effects of acute salt loading in young normotensive individuals. Hypertension. 2008;51:1525–30. 18458163 10.1161/HYPERTENSIONAHA.108.109868

[52] Mensink RP, Zock PL, Kester AD, Katan MB. Effects of dietary fatty acids and carbohydrates on the ratio of serum total to HDL cholesterol and on serum lipids and apolipoproteins: a meta-analysis of 60 controlled trials. Am J Clin Nutr. 2003;77:1146–55. 12716665 10.1093/ajcn/77.5.1146

[53] Martínez Leo EE, Peñafiel AM, Hernández Escalante VM, Cabrera Araujo ZM. Ultra-processed diet, systemic oxidative stress, and breach of immunologic tolerance. Nutrition. 2021;91-92:111419. 34399404 10.1016/j.nut.2021.111419

[54] Coppola S, Paparo L, Trinchese G, Rivieri AM, Masino A, De Giovanni Di Santa Severina AF, et al. Increased dietary intake of ultraprocessed foods and mitochondrial metabolism alterations in pediatric obesity. Sci Rep. 2023;13:12609. 37537205 10.1038/s41598-023-39566-9 PMC10400566

[55] Zeinalabedini M, Nasli-Esfahani E, Esmaillzadeh A, Azadbakht L. How is healthy eating index-2015 related to risk factors for cardiovascular disease in patients with type 2 diabetes. Front Nutr. 2023;10:1201010. 37305085 10.3389/fnut.2023.1201010 PMC10248502

[56] Krebs-Smith SM, Pannucci TE, Subar AF, Kirkpatrick SI, Lerman JL, Tooze JA, et al. Update of the healthy eating index: HEI-2015. J Acad Nutr Diet. 2018;118:1591–602. 30146071 10.1016/j.jand.2018.05.021 PMC6719291

[57] Reedy J, Lerman JL, Krebs-Smith SM, Kirkpatrick SI, Pannucci TE, Wilson MM, et al. Evaluation of the healthy eating index-2015. J Acad Nutr Diet. 2018;118:1622–33. 30146073 10.1016/j.jand.2018.05.019 PMC6718954

[58] Hosseininasab D, Shiraseb F, Noori S, Jamili S, Mazaheri-Eftekhar F, Dehghan M, et al. The relationship between ultra-processed food intake and cardiometabolic risk factors in overweight and obese women: a cross-sectional study. Front Nutr. 2022;9:945591. 36017229 10.3389/fnut.2022.945591 PMC9396040

[59] Nouri M, Eskandarzadeh S, Makhtoomi M, Rajabzadeh-Dehkordi M, Omidbeigi N, Najafi M, et al. Association between ultra-processed foods intake with lipid profile: a cross-sectional study. Sci Rep. 2023;13:7258. 37142735 10.1038/s41598-023-34451-x PMC10160124

