# Supplementary material for: Association of ultra-processed food consumption with cardiovascular risk factors among patients with type-2 diabetes mellitus
Source: Nutr Diabetes. 2024 Oct 22;14:89. doi: 10.1038/s41387-024-00337-8 (PMC11494205; doi:10.1038/s41387-024-00337-8)
Supplement: Supplementary file 1 — Multivariable-adjusted odds ratio novel CVD risk factors and anthropometrics across tertiles of UPFs consumption [file 41387_2024_337_MOESM1_ESM.docx]

**Supplementary Table 1: Multivariable-adjusted odds ratio novel CVD risk factors and anthropometrics across tertiles of UPFs^1^ consumption^2^**

| Outcome | UPF consumption | | | P-trend ^a^ |
| --- | --- | --- | --- | --- |
|  | **Tertile 1 (lowest)**  **(N = 163)** | **Tertile 2**  **(N = 163)** | **Tertile 3 (highest)**  **(N = 163)** |  |
| CRI-I | | | | |
| Crude ^b^ | 1 | 1.19 (0.74-1.92) | 1.83 (1.15–2.91) | 0.01 |
| Model 1^c^ | 1 | 0.95 (0.61-1.49) | 0.93 (0.56-1.54) | 0.06 |
| Model 2^d^ | 1 | 0.76 (0.48-1.22) | 0.64 (0.37-1.10) | 0.78 |
| CRI-II | | | | |
| Crude | 1 | 1 (0.62-1.60) | 1.42 (0.90-2.25) | 0.12 |
| Model 1 | 1 | 0.90 (0.56-1.46) | 1.08 (0.64-1.84) | 0.76 |
| Model 2 | 1 | 0.77 (0.47-1.28) | 0.82 (0.46-1.45) | 0.49 |
| CI | | | | |
| Crude | 1 | 1.06 (0.66-1.70) | 1.63 (1.02-2.58) | 0.03 |
| Model 1 | 1 | 0.94 (0.58-1.54) | 1.20 (0.71-2.05) | 0.48 |
| Model 2 | 1 | 0.85 (0.51-1.40) | 1.00 (0.57-1.77) | 0.99 |
| AIP | | | | |
| Crude | 1 | 0.91 (0.57-1.46) | 1.11 (0.70-1.76) | 0.63 |
| Model 1 | 1 | 0.92 (0.57-1.49) | 1.14 (0.67-1.92) | 0.64 |
| Model 2 | 1 | 0.72 (0.44-1.19) | 0.83 (0.47-1.46) | 0.49 |
| LAP | | | | |
| Crude | 1 | 1.54 (0.96-2.47) | 1.71 (1.07-2.74) | 0.026 |
| Model 1 | 1 | 1.61 (0.99-2.62) | 1.93 (1.13-3.31) | 0.015 |
| Model 2 | 1 | 1.57 (0.95-2.61) | 2.20 (1.23-3.93) | 0.008 |
| AVI |  |  |  |  |
| Crude | 1 | 0.97 (0.60-1.55) | 1.41 (0.89-2.23) | 0.13 |
| Model 1 | 1 | 1.06 (0.66-1.72) | 1.82 (1.07-3.08) | 0.02 |
| Model 2 | 1 | 1.04 (0.63-1.72) | 2.02 (1.14-3.58) | 0.01 |
| BRI |  |  |  |  |
| Crude | 1 | 0.97 (0.62-1.52) | 0.51 (0.31-0.82) | 0.007 |
| Model 1 | 1 | 1.17 (0.73-1.86) | 0.80 (0.46-1.39) | 0.49 |
| Model 2 | 1 | 1.96 (1.10-3.48) | 2.14 (1.04-4.40) | 0.02 |
| ABSI |  |  |  |  |
| Crude | 1 | 1.24 (0.78-1.95) | 0.89 (0.55-1.42) | 0.63 |
| Model 1 | 1 | 1.45 (0.90-2.33) | 1.32 (0.77-2.28) | 0.27 |
| Model 2 | 1 | 1.70 (1.02-2.84) | 2.16 (1.18-3.97) | 0.01 |

Abbreviations: ABSI, A body shape index; AVI, Abdominal volume index; BRI, Body roundness index; AIP, Atherogenic index of plasma; CRI-1, Castelli risk index-1; CRI-2; Castelli risk index 2; LAP, Lipid accumulation product

^1^Ultra-processed foods (UPF) defined based on NOVA classification for food processing

^2^ All values are odds ratios and 95% confidence intervals

^a^ Calculated using logistic regression; ^b^: Crude: Not adjusted for any variables; ^c^: Model adjusted for energy intake;
^d^ Model adjusted for energy intake plus socioeconomic status, age, sex, and smoking
